# Supplementary material for: NF-kB Regulation and the Chaperone System Mediate Restorative Effects of the Probiotic Lactobacillus fermentum LF31 in the Small Intestine and Cerebellum of Mice with Ethanol-Induced Damage
Source: Biology (Basel). 2023 Nov 1;12(11):1394. doi: 10.3390/biology12111394 (PMC10669058; doi:10.3390/biology12111394)
Supplement: Supplementary file 1 [file biology-12-01394-s001.zip › biology-2657375-supplementary.pdf]

NF-kB regulation and the chaperone system mediate restorative effects of the probiotic *Lactobacillus fermentum* LF31 in the small intestine and cerebellum of mice with ethanol-induced damage

Letizia Paladino, Francesca Rappa, Rosario Barone, Filippo Macaluso, Francesco Paolo Zummo, Sabrina David, Marta Anna Szychlinska, Fabio Bucchieri, Everly Conway de Macario, Alberto J L Macario, Francesco Cappello, Antonella Marino Gammazza

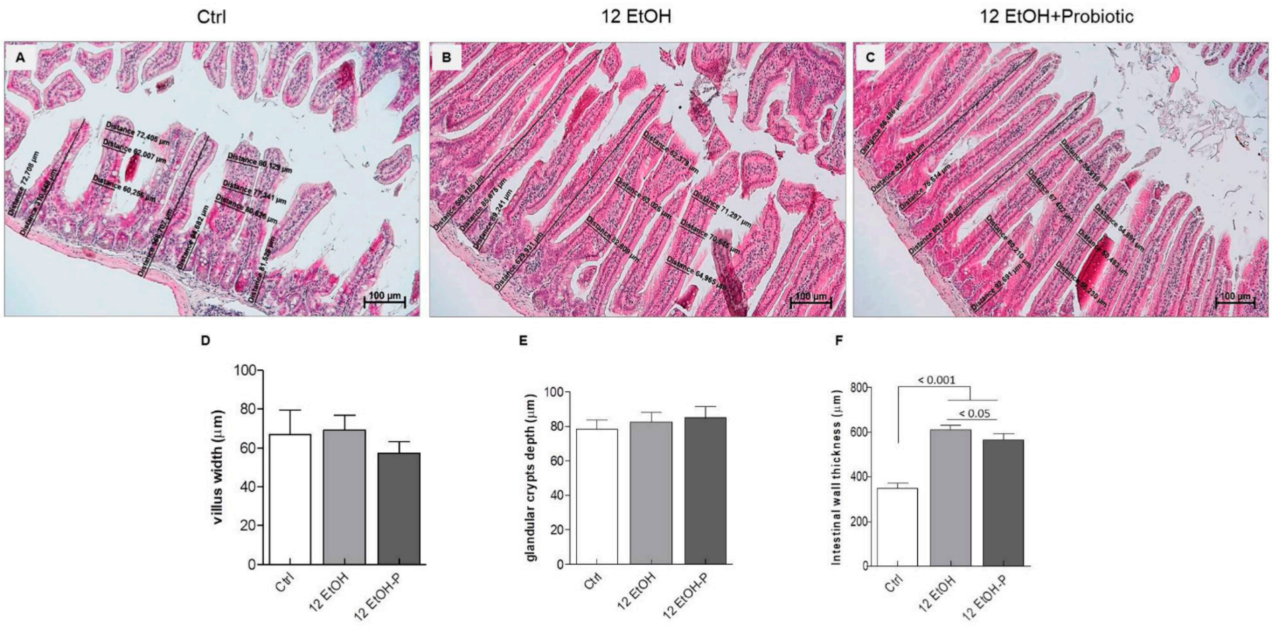

Figure S1: Small intestinal histomorphometry. The morphological analysis performed on the mucosa of the jejunum, stained with H&E, was conducted to evaluate the width of the intestinal villi, the depth of the glandular crypts, and the thickness of the entire intestinal wall of mice fed with a standard diet (Ctrl, **A**), ethanol (12 EtOH, **B**), and ethanol and *L. fermentum* LF31 diets (12 EtOH+Probiotic, **C**). The data obtained show no statistically significant differences in the evaluations of the width of the villi and the depth of the glandular crypts (**D** and **E**, respectively). On the other hand, the intestinal wall thickness, as a consequence of the difference in villus length, was significantly greater in the 12 EtOH and 12 EtOH+Probiotic groups than in the control group ($p<0.001$) and between the 12 EtOH and 12 EtOH+Probiotic groups ($p<0.05$) (**G**).
